# The acute effects on duodenal gene expression in healthy men following consumption of a low-fat meal enriched with theobromine or fat

**DOI:** 10.1038/s41598-018-20068-y

**Published:** 2018-01-26

**Authors:** Lotte Smolders, Ronald P. Mensink, Mark V. Boekschoten, Rogier J. J. de Ridder, Jogchum Plat

**Affiliations:** 1 Department of Human Biology and Movement Sciences, School of Nutrition and Translational Research in Metabolism (NUTRIM), Maastricht University Medical Center, Maastricht, PO Box 616, 6200 MD The Netherlands; 2 Nutrition, Metabolism and Genomics Group, Wageningen University, Wageningen, The Netherlands; 3 Division of Gastroenterology and Hepatology, Maastricht University Medical Center, Maastricht, The Netherlands

## Abstract

Increasing apoA-I synthesis may improve HDL functionality and lower CVD risk. As theobromine and fat increase fasting apoA-I concentrations, and the intestine is involved in apoA-I production, the acute effects of both were studied on duodenal gene transcription to better understand underlying mechanisms. In this crossover study, 8 healthy men received once a low fat (LF) meal, a LF meal plus theobromine (850 mg), or a high fat (HF) meal. Five hours after meal intake duodenal biopsies were taken for microarray analysis. Theobromine and HF consumption did not change duodenal apoA-I expression. Theobromine did not change gene expression related to lipid and cholesterol metabolism, whereas those related to glycogen/glucose breakdown were downregulated. HF consumption increased gene expression related to lipid and cholesterol uptake and transport, and to glucose storage, while it decreased those related to glucose uptake. Furthermore, genes related to inflammation were upregulated, but inflammation markers in plasma were not changed. In healthy men, acute theobromine and fat consumption did not change duodenal apoA-I mRNA, but inhibited expression of genes related to glucose metabolism. Furthermore, HF intake activated in the duodenum expression of genes related to lipid and cholesterol metabolism and to inflammation.

## Introduction

Over the past decade, interventions aiming to increase serum high-density lipoprotein (HDL) cholesterol (HDL-C) concentrations to reduce the risk of cardiovascular disease (CVD) have not been successful. Recent insights, however, suggest that improving HDL functionality will more likely lower CVD risk than simply elevating circulating serum HDL-C concentrations^1^. In this respect, increasing serum apolipoprotein A-I (apoA-I) synthesis may be a promising approach, as serum apoA-I concentrations correlate with *in vitro* cholesterol efflux, a measure of HDL functionality^2–4^. For this, various pharmaceutical approaches are currently explored^5^. However, as a preventive strategy at a population level, dietary strategies are more useful. Unfortunately, the number of (novel) dietary components that increase apoA-I production is limited. Theobromine, a component from cocoa, has been reported to increase fasting serum apoA-I concentrations^6^. Mechanisms explaining the effects of theobromine on fasting serum apoA-I concentrations are however not clear. Since apoA-I is produced in enterocytes and hepatocytes^7^, and theobromine is absorbed in the small intestine^8,9^, we were interested in exploring the effects of acute theobromine consumption on gene expression in postprandial human duodenal biopsies, following theobromine intake. Duodenal biopsies were used, because apoA-I secretion is higher in the duodenum as compared with other parts of the intestine^10^.

Except theobromine, also exchanging carbohydrates for fatty acids increases fasting serum apoA-I concentrations^11,12^. Although the intestine is strongly involved in dietary lipid handling, the effects of fat intake on duodenal gene expression profiles have not been studied. Only a limited number of studies compared the effects of a low-fat/high-carbohydrate (LF) with a high-fat/low-carbohydrate (HF) meal on gene expression profiles, and so far only in human muscle biopsies^13–15^ and peripheral blood mononuclear cells (PBMCs)^16^. We therefore used a nutrigenomic approach to analyze differences in gene expression in human duodenal biopsies after adding 850 mg of theobromine to a LF meal, and after comparing HF with LF meal consumption to better understand effects of dietary theobromine and fat on duodenal apoA-I transcription and related pathways.

## Results

### Subject characteristics

All ten men completed the study. However, results of one man were excluded due to protocol violation, as he appeared not to be in fasting condition at start of one of the test days. Samples from a second subject were excluded due to technical issues during microarray analysis. Baseline characteristics of the final eight subjects are shown in Table 1.

**Table 1:** Baseline characteristics of the participants who completed the study^1^.

	Mean ± SD
Age (years)	38 ± 15
BMI (kg/m^2^)	24.3 ± 2.0
Serum total cholesterol (mmol/L)	5.2 ± 0.9
Plasma glucose (mmol/L)	5.1 ± 0.3
Systolic blood pressure (mmHg)	130 ± 18
Diastolic blood pressure (mmHg)	79 ± 14

^1^Values are mean ± SD. *n* = 8.

### Microarray analysis

From the 19697 genes present on the microarray, 10506 genes were expressed in the duodenum (expression value > 20 and > 5 probes per gene on the array). In comparison to the LF shake, 113 and 286 genes were differentially expressed after adding theobromine to the LF diet (LF-TB) and comparing it with the HF shake, respectively (Supplementary data Fig. 1). Twenty-three of these differentially expressed genes overlapped, i.e. the expression was significantly changed into the same direction after both the LF-TB and the HF interventions as compared with the LF diet. These 23 genes were SCN3B, PCDH11Y, NELL2, LYPD6B, ZNF485, FBXL16, PLA1A, ZBTB16, UPK2, DKK4, TIAM2, CKLF, SPRR1A, C12orf74, MTNR1A, KCNS3, MBOAT2, VSIG8, TEN1-CDK3, PDE10A, LRP12, TAS2R3 and RAD51AP1. So far, none of these 23 genes was described in relation to apoA-I transcriptional regulation. Unfortunately, apoA-I gene expression did not change when comparing the LF-TB meal vs. the LF meal (FC = 1.02, P = 0.758) or the HF meal vs. the LF meal (FC = 1.07, P = 0.310). Still, these 23 genes are potentially interesting targets to consider in the context of apoA-I transcription.

**Figure 1:**
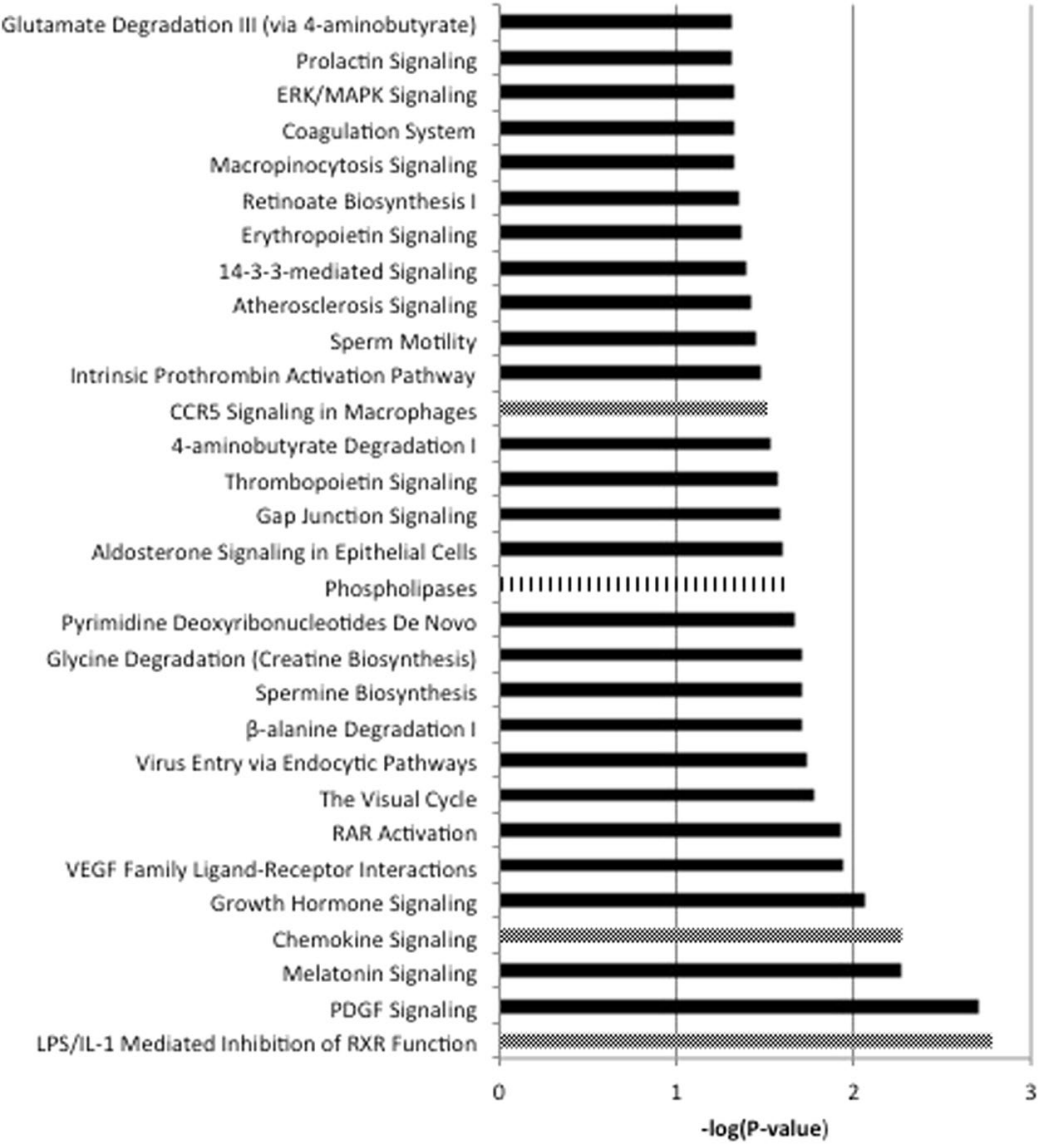
Significantly different pathways after adding 850 mg of theobromine (TB) to the low-fat/high-carbohydrate (LF) diet. Dotted bars are pathways involved in the immune system, lined bars are pathways involved in cholesterol, lipid or glucose metabolism (*n* = 8).

Next, gene expression profiles were further explored, to better understand the effects of adding theobromine to a LF diet or comparing a HF with a LF diet on duodenal gene expression. First, IPA was used to look at significantly changed pathways. Then, two more in depth analytical procedures were conducted, i.e. the Upstream Regulator Analysis and GSEA.

### Adding TB to a LF meal

#### Ingenuity Pathway Analysis

Thirty pathways were differentially regulated by theobromine consumption (Fig. 1). None of the pathways was related to cholesterol, lipid or glucose metabolism.

#### Upstream Regulator Analysis

Adding TB to the LF meal changed the activation of 29 transcriptional regulators (Supplementary data, Table 1). Three of these upstream regulators, which were related to glucose metabolism, were inhibited. Further, the other identified upstream regulators were not linked to the lipid and cholesterol metabolism (Table 2).

**Table 2:** Inhibited or activated upstream regulators after adding 850 mg of theobromine (TB) to a low-fat/high-carbohydrate (LF) meal or after comparing high-fat/low-carbohydrate (HF) with LF consumption functioning in lipid, cholesterol or glucose metabolism or the immune system (*n* = 8).

Upstream regulator	Activation Z-score	Function (gene card ref)	Comparison
Ins1	−3.02	Decreases blood glucose	LF-TB vs. LF
Insulin	−2.08	Decreases blood glucose	LF-TB vs. LF
INS	−1.95	Decreases blood glucose	LF-TB vs. LF
INSIG2	−1.95	Feedback control of cholesterol synthesis	HF vs. LF
APOE	−1.73	Main lipoprotein on chylomicrons	HF vs. LF
ACOX1	−1.63	Fatty acid β-oxidation pathway	HF vs. LF
SREBF2	1.93	Lipid homeostasis	HF vs. LF
PPARα	1.97	Transcription factor in lipid and cholesterol metabolism	HF vs. LF
FABP2	2.00	Uptake and transport of long chain fatty acids involved in TAG-rich lipoprotein synthesis	HF vs. LF
SREBF1	2.27	Transcription factor which regulates lipid homeostasis	HF vs. LF
GCG	−2.42	Proprotein for glucagon	HF vs. LF
GCK	−1.98	Enzyme functioning in glucose utilization	HF vs. LF
Gsk3	1.98	Glycogen synthesis	HF vs. LF
TNFSF12	1.65	Activation of NFκB, inducer of proinflammatory cytokines	HF vs. LF
IL12	1.90	Proinflammatory cytokine	HF vs. LF
IL2	1.92	Proinflammatory cytokine	HF vs. LF
CD5	1.98	Receptor in the regulation of T-lymphocyte proliferation	HF vs. LF
CCL5	1.98	Chemoattractant for monocytes, T-lymphocytes, eosinophils	HF vs. LF
TNF	1.99	Survival, proliferation and differentiation of monocytes and macrophages	HF vs. LF
RELA	2.02	Subunit NFκB	HF vs. LF
MYD88	2.24	Innate immune response	HF vs. LF
IL1A	2.31	Proinflammatory cytokine	HF vs. LF
TNFSF11	2.73	T-lymphocyte dependent immune response	HF vs. LF
IL1B	2.82	Proinflammatory cytokine	HF vs. LF

#### Gene Set Enrichment Analysis

In the GSEA, 6 gene sets were upregulated and 2 gene sets were downregulated (Supplementary data, Table 2). One of the downregulated set was glucose metabolism (Table 3).

**Table 3:** Significantly inhibited or activated gene sets after adding 850 mg of theobromine (TB) to a low-fat/high-carbohydrate (LF) meal or after comparing high-fat/low-carbohydrate (HF) with LF meal consumption functioning in lipid, cholesterol or glucose metabolism or the immune system (*n* = 8).

Name gene set	NES	FDR q-val	Comparison
Glucose metabolism	−1.96	0.137	LF-TB vs. LF
Regulation of beta cell development	1.96	0.119	LF-TB vs. LF
Fatty acid beta oxidation	2.09	0.025	HF vs. LF
Regulation of lipid metabolism by PPARα	1.93	0.057	HF vs. LF
PPARα activates gene expression	1.92	0.058	HF vs. LF
Mitochondrial long chain fatty acid β oxidation	1.88	0.075	HF vs. LF
TAG synthesis	1.82	0.106	HF vs. LF
Regulation of lipid metabolism by PPARα	1.81	0.104	HF vs. LF
PPARα signaling pathway	1.80	0.104	HF vs. LF
PPARα targets	1.80	0.101	HF vs. LF
Statin pathway	1.67	0.144	HF vs. LF
SREBP signaling	1.62	0.159	HF vs. LF
Lipid digestion, mobilization and transport	1.60	0.171	HF vs. LF
Fatty acid TAG and ketone body metabolism	1.60	0.167	HF vs. LF
Lipid digestion, mobilization and transport	1.56	0.184	HF vs. LF
Metabolism of steroid hormones and Vit D	1.58	0.175	HF vs. LF
Steroid hormones	1.57	0.178	HF vs. LF
Regulation of cholesterol synthesis by SREBP SREBF	1.56	0.181	HF vs. LF
Lipid digestion, mobilization and transport	1.55	0.183	HF vs. LF
Cholesterol biosynthesis	1.53	0.199	HF vs. LF
Glucose metabolism	−2.29	0.001	HF vs. LF
Metabolism of carbohydrates	−2.17	0.006	HF vs. LF
Gluconeogenesis	−1.84	0.074	HF vs. LF
Hexose transport	−1.83	0.073	HF vs. LF
Carbohydrates digestion and absorption	−1.83	0.070	HF vs. LF
Glycogen metabolism	−1.78	0.064	HF vs. LF
Starch and sucrose metabolism	−1.77	0.058	HF vs. LF
Hexose transport	−1.67	0.088	HF vs. LF
Pancreatic secretion	−1.61	0.129	HF vs. LF
Insulin signalling pathway	−1.59	0.136	HF vs. LF
Glycolysis and gluconeogenesis	−1.47	0.193	HF vs. LF
Type II diabetes mellitus	−1.47	0.196	HF vs. LF
Glycogen storage diseases	−1.46	0.198	HF vs. LF
Glucose transport	−1.45	0.196	HF vs. LF
Chemokine receptors bind chemokines	2.08	0.020	HF vs. LF
Cell adhesion molecules CAMS	1.82	0.113	HF vs. LF
Cytokine cytokine receptor interaction	1.82	0.110	HF vs. LF
Cytokine and inflammatory response	1.80	0.103	HF vs. LF
Intestinal immune network for IGA production	1.79	0.106	HF vs. LF
RIP mediated NFκB activation via ZBP1	1.78	0.106	HF vs. LF
ZBP1 DAI mediated induction of type I IFNS	1.78	0.101	HF vs. LF
IL1R pathway	1.78	0.089	HF vs. LF
Rheumatoid arthritis	1.77	0.101	HF vs. LF
NKT pathway	1.74	0.110	HF vs. LF
TNF signaling pathway	1.73	0.112	HF vs. LF
Staphylococcus aureus infection	1.71	0.126	HF vs. LF
NFκB signaling pathway	1.65	0.176	HF vs. LF
TOPB1 pathway	1.60	0.168	HF vs. LF
NTHI pathway	1.59	0.173	HF vs. LF
Inflammatory response pathway	1.59	0.167	HF vs. LF
TNFR2 pathway	1.57	0.180	HF vs. LF
Human complement system	1.55	0.184	HF vs. LF

#### Overall gene expression pattern after adding TB to a LF meal

Overall, adding TB to a LF meal did not change the expression of genes related to lipid and cholesterol metabolism, whereas, expression of a number of genes related to glycogen and glucose breakdown were downregulated.

### Comparing HF with LF meal consumption

#### Ingenuity Pathway Analysis

**Figure 2:**
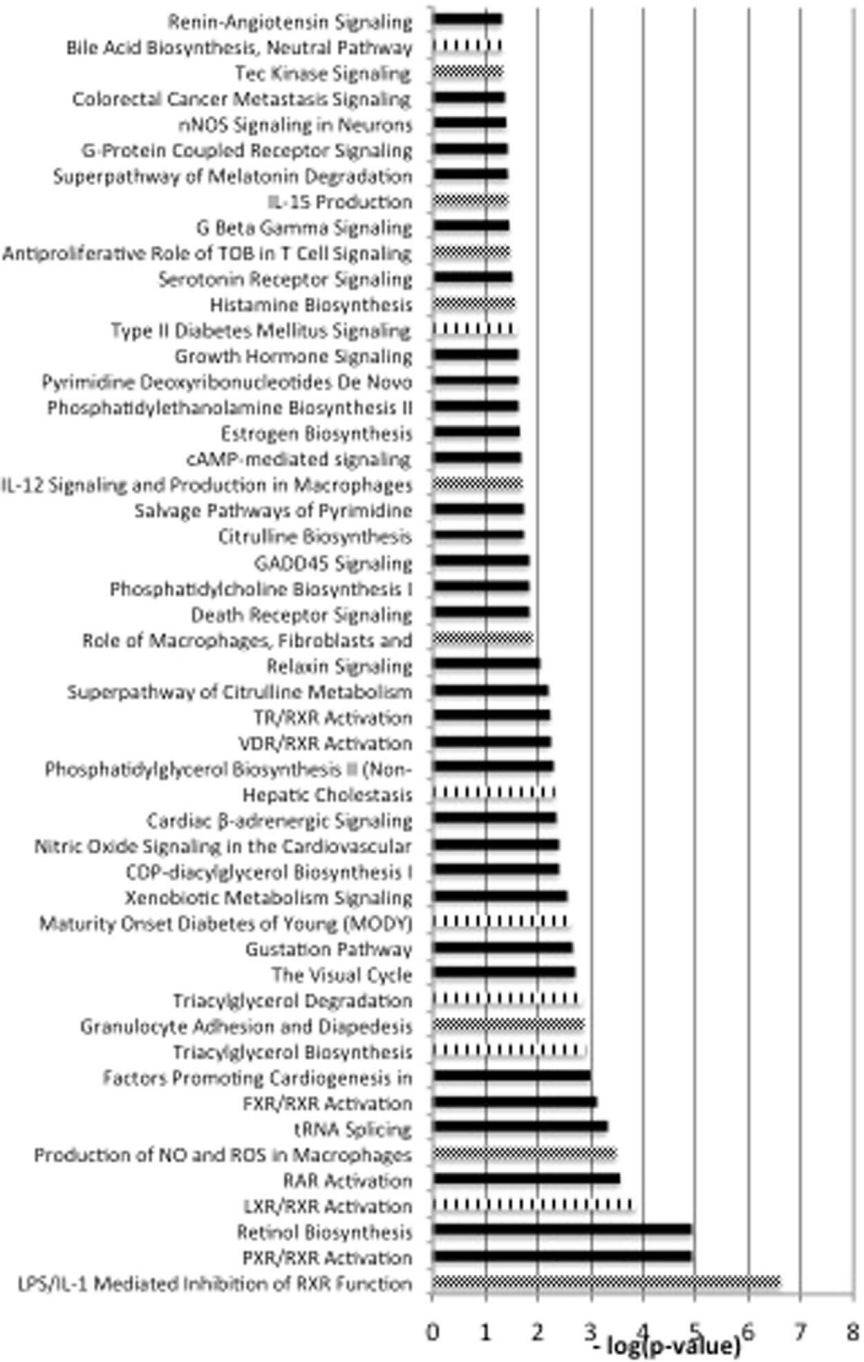
Significantly different pathways comparing a high-fat/low-carbohydrate (HF) with a low-fat/high-carbohydrate (LF) diet. Dotted bars are pathways involved in the immune system, lined bars are pathways involved in cholesterol, lipid or glucose metabolism (*n* = 8).

Fifty pathways were differentially regulated after intake of the HF meal as compared with the LF meal. Seven of these pathways were linked to cholesterol, lipid or glucose metabolism, such as LXR/RXR activation and triacylglycerol degradation/biosynthesis. Also 9 changed pathways were related to the immune response, including the production of IL12 and IL15 and the production of NO and ROS in macrophages (Fig. 2).

#### Upstream Regulator Analysis

The activation of 113 transcriptional regulators was changed (Supplementary data, Table 3). After HF consumption regulators involved in lipid uptake and transport such as SREBF2, PPARα and FABP2 were activated, while regulators involved in fatty acid breakdown such as ACOX1 were inhibited. Furthermore, regulators involved in glucose uptake, including GCG and GCK were inhibited and regulators involved in glucose storage, such as Gsk3 were activated. Finally, HF meal consumption activated 11 proinflammatory regulators involved in the immune response: TNFSF12, IL12, IL2, CCL5, TNF, CD5, RELA, MYD88, TNFSF11, IL1A and IL1B (Table 2).

#### Gene Set Enrichment Analysis

**Figure 3:**
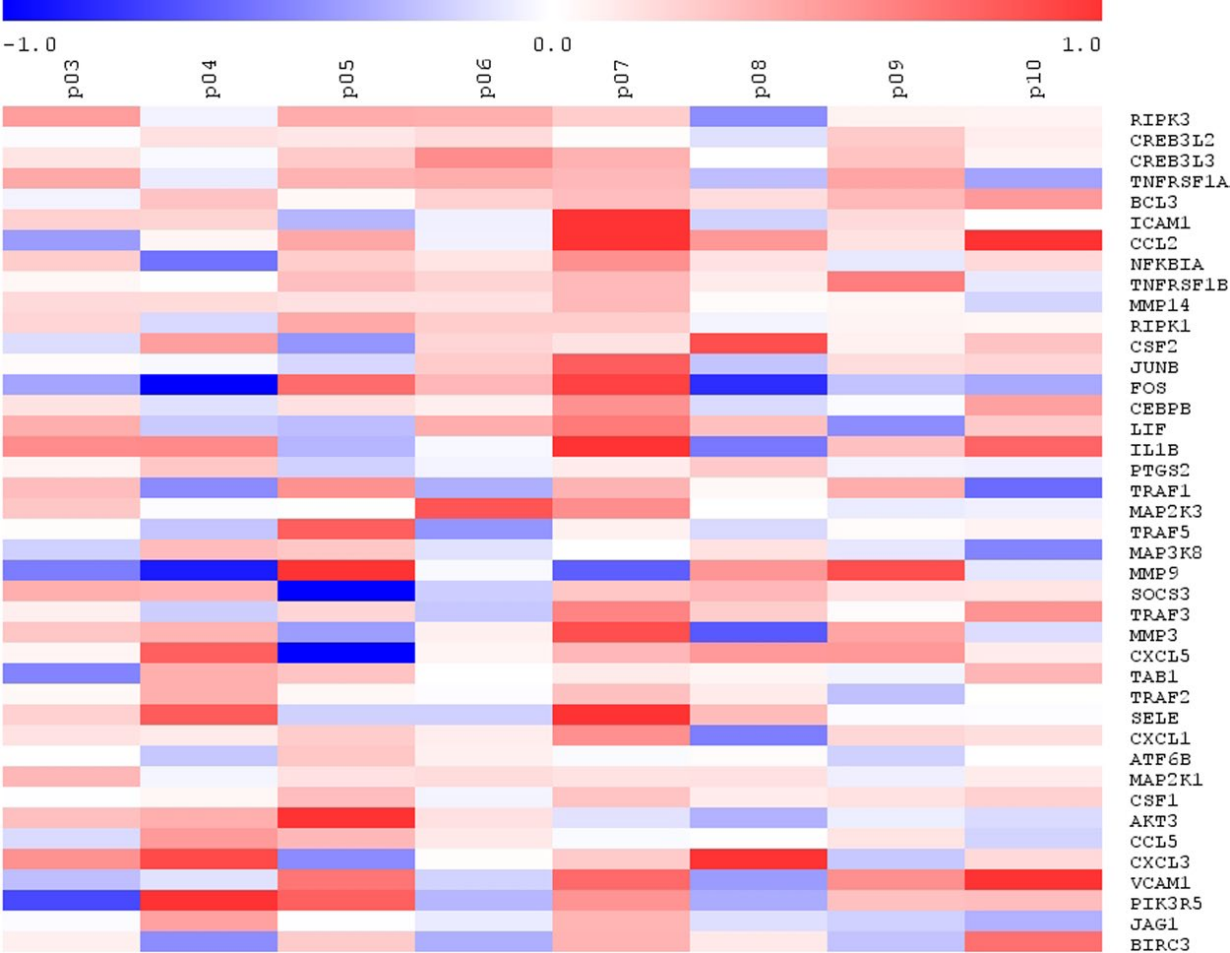
Heatmap of the of the TNF signaling pathway from the GSEA results after the consumption of an acute high-fat/low carbohydrate (HF) vs. low-fat/high-carbohydrate (LF) meal (*n* = 8).

GSEA showed 83 upregulated gene sets and 176 downregulated gene sets after HF meal consumption (Supplementary data, Table 4). Seventeen gene sets involved in lipid and cholesterol metabolism were upregulated. These gene sets suggested an increased activity of PPARα, fatty acid oxidation, fatty acid, triacylglycerol (TAG) and lipoprotein metabolism, lipid digestion, mobilization and transport, cholesterol synthesis, steroid hormone metabolism and SREBP signaling. Furthermore, the 13 gene sets gene sets that were downregulated were involved in glucose and carbohydrate metabolism, suggesting an inhibited glucose and carbohydrate metabolism, gluconeogenesis and insulin signaling. Finally, 19 upregulated gene sets were involved in the immune system, including chemokine and cytokine activities, NFκB pathway, complement system and TNF signaling pathway (Table 3 and Fig. 3).

#### Overall gene expression pattern comparing HF with LF consumption

HF consumption increased the expression of genes related to lipid and cholesterol uptake and transport and glucose storage, while it decreased the expression of genes related glucose uptake. Furthermore, all three approaches showed upregulated expression of genes related to the immune response and inflammation.

### Markers for inflammation, the acute phase response, endothelial function and intestinal damage

Since the three analytical approaches consistently showed differences in the expression profiles of genes involved in the cytokine and inflammatory responses after HF compared with LF meal consumption, a panel of plasma markers for inflammation and acute phase responses were analyzed to evaluate whether this acute change in inflammatory gene expression in the duodenum was also transferred into the circulation.

**Figure 4:**
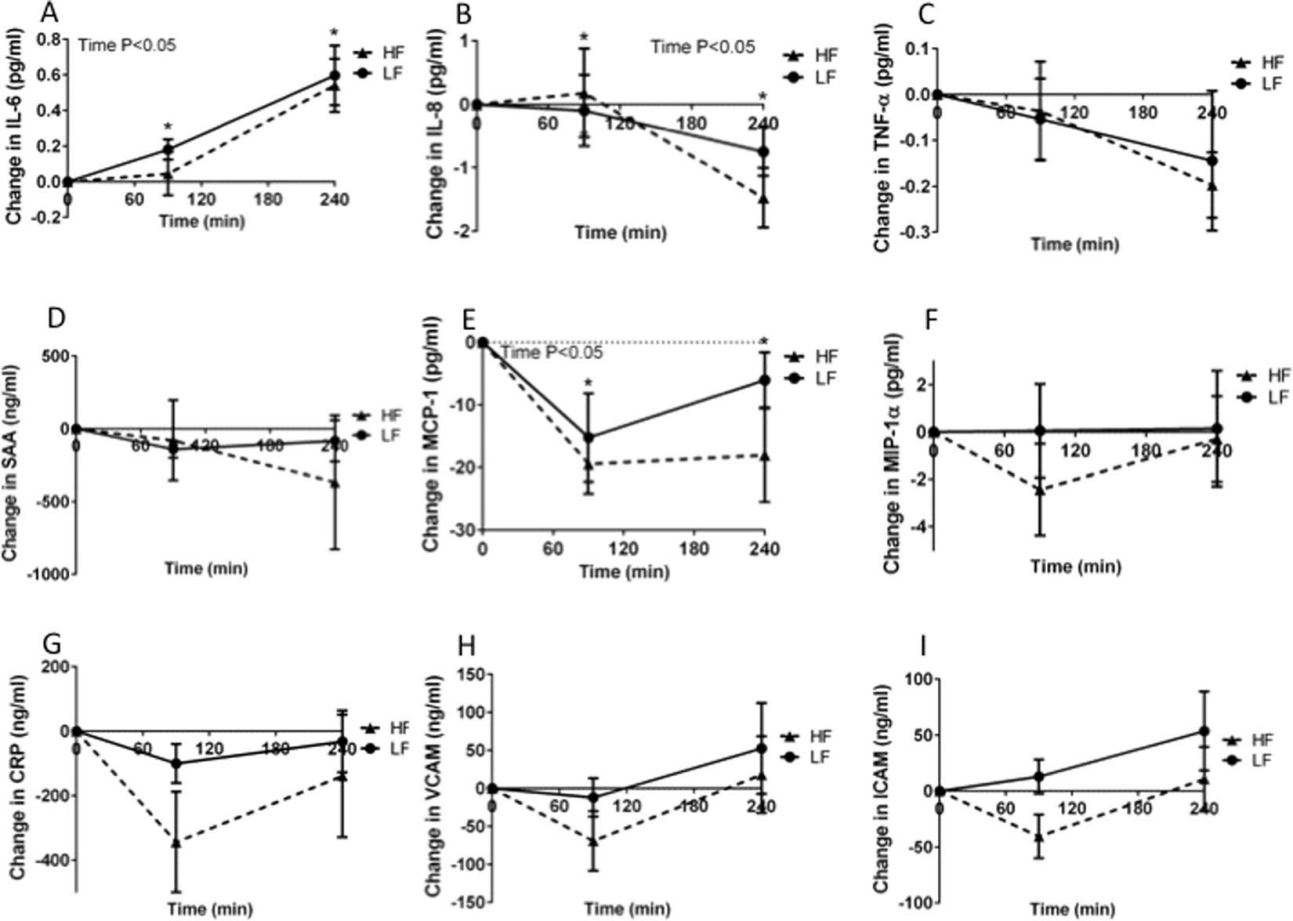
Change in (**A**), interleukin 6 (IL-6) (**B**), interleukin 8 (IL-8) (**C**), tumor necrosis factor alpha (TNF-α) (**D**), serum amyloid A (SAA) (**E**), monocyte chemoattractant protein-1 (MCP-1) (**F**), macrophage inflammatory protein 1 a (MIP-1a) (**G**), high sensitive C-reactive protein (CRP) (**H**), vascular cell adhesion protein (VCAM) and (**I**), intercellular adhesion molecule (ICAM) after acute low-fat/high-carbohydrate (LF, black line, squares) and acute high-fat/low carbohydrate (HF, dotted line, triangles) meal consumption^1^. ^1^Values are mean ± SD. *n* = 8.

**Figure 5:**
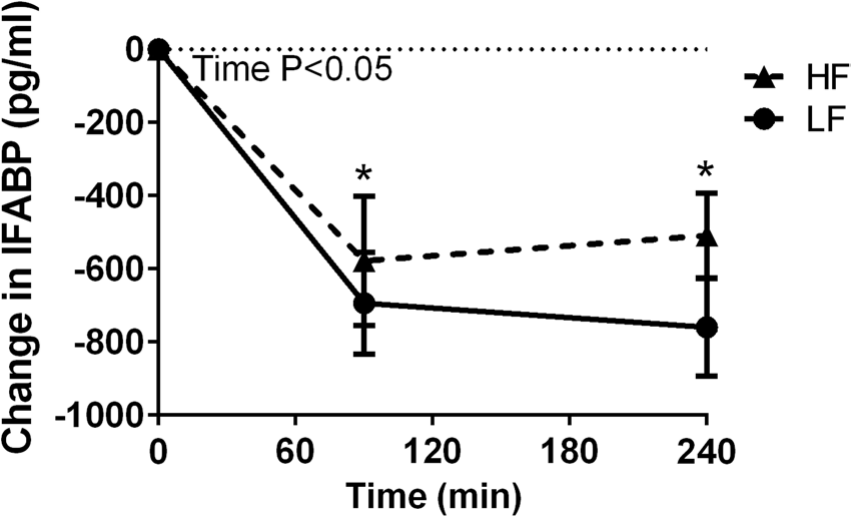
Change in intestinal fatty acid-binding protein (IFABP) after acute low-fat/high-carbohydrate (LF, black line, squares) and acute high-fat/low-carbohydrate (HF, dotted line, triangles) meal consumption. ^1^Values are mean ± SD. *n* = 8.

During the postprandial phase, IL-6 concentrations significantly increased over time (P < 0.001), while IL-8, MCP-1 and IFABP concentrations significantly decreased over time (P = 0.006, P < 0.001 and P < 0.001, respectively). Concentrations of all other markers for inflammation and the acute phase response, as well as endothelial function did not change over time (Fig. 4). Moreover, no differences in postprandial changes in markers for inflammation, acute phase response and endothelial function between the HF and LF meals were observed (Fig. 4). Finally, circulating levels of IFABP were measured to evaluate the potential effects of the HF and LF meal on intestinal damage. However, also IFABP concentrations were not different between HF and LF meal consumption (Fig. 5).

## Discussion

It has been shown that consuming either theobromine^6^ or fat^12^ increased fasting serum apoA-I concentrations. To better understand the potential role of the intestine in the mechanisms underlying these effects on apoA-I concentrations, as well as to explore effects on gene expression profiles in general, we examined in the present randomized, double-blind, controlled study the acute effects of theobromine and of HF consumption on duodenal gene expression. Unfortunately, both theobromine and HF consumption did not change duodenal apoA-I gene expression. For this apparently discrepant result as compared to the assumed effects on serum apoA-I concentrations, there are at least four explanations. First, theobromine and fat only change duodenal apoA-I mRNA expression after longer-term consumption. Second, postprandial effects on apoA-I transcription are not evident within a time period of 5 hours. Third, theobromine and fat regulate apoA-I metabolism not at a transcriptional level in the duodenum, but only in the liver. However, this explanation is less likely, as postprandial serum apoA-I concentrations did also not change after theobromine and HF intake^17^. Fourth, theobromine and fat do not change apoA-I transcription but increases serum apoA-I concentrations via other mechanisms, e.g. a reduction in apoA-I clearance. Indeed, replacement of 13 energy % of carbohydrates for MUFA, which elevated apoA-I concentrations, decreased apoA-I fractional catabolic rate, while it did not change apoA-I production rate^18^. It should also be noted, that unexpectedly we could recently not confirm in a long-term study the effects of theobromine on apoA-I^19^, as reported by Neufingerl *et al*.^6^.

Except for evaluating effects on duodenal apoA-I mRNA expression, we also explored changes in gene expression profiles in general. In this context, acute theobromine consumption did not change the expression of genes related to lipid and cholesterol metabolism. This observation is in agreement with the earlier reported lack of acute effects in changing postprandial serum concentrations of apoB100, apolipoprotein B48, TAG and free fatty acids^17^, but not with the results of 4-weeks of theobromine consumption, which improved fasting lipid concentrations^6,19^. Except for apoB48, which is only synthesized in the intestine, the same considerations as those for apoA-I can be used to explain the lack of effects. Moreover, GSE analysis showed that theobromine inhibited duodenal expression of gene sets involved in glucose metabolism, suggesting a lower glycogen and glucose breakdown. Moreover, using the upstream regulator analysis, we identified downregulation of three factors (Ins1, Insulin and INS) that have a functional role in lowering plasma glucose concentrations. Thus, these changes are expected to translate into higher plasma glucose concentrations. Indeed, we have earlier reported increased postprandial glucose responses after 4-week TB-consumption^19^. Though suggestive, it is however unclear how and to what extent these decreases in gene expression relate to changes in glucose metabolism in the duodenum and to changes in plasma glucose and insulin responses. Besides these effects of theobromine on expression of genes involved in glucose metabolism, there were no other consistent clusters of metabolic processes that seemed to be sensitive to theobromine consumption. However, it should be noted that only effects of theobromine on a LF background diet were studied. Therefore, it cannot be excluded that effects of theobromine on a HF background would have been different.

Besides theobromine, also the acute effects of switching from a LF to HF diet on duodenal gene expression profiles were examined. Surprisingly, in humans these type of analysis during the postprandial phase has been done in muscle biopsies and circulating leukocyte fractions [13–16], but hardly in the small intestine, which plays a major role in TAG and cholesterol absorption and transport^20^. As expected, the HF meal increased postprandial serum TAG concentrations^17^, and upregulated genes related to lipid and cholesterol uptake and transport when compared with the LF meal. The upregulation in LXR/RXR pathway expression, triacylglycerol degradation and biosynthesis, as well as bile acid biosynthesis pathways, as observed in the GSE analysis, are illustrative. The changes in the expression of transcription factors, such as downregulation in INSIG2 (cholesterol biosynthesis), and upregulation in SREBF2 & SREBF1 (lipid homeostasis), and PPARα (lipid and cholesterol metabolism), are generally in line with these pathways. This can be explained by the higher amount of cholesterol and lipids that are present in the intestine after HF meal consumption, as these lipids need to be taken up and must be transported to other tissue. In agreement, in the intestines of mice, genes related to lipid metabolism were activated when long-term HF intake was compared with LF intake^21^. Also in human muscle biopsies, expression of genes functioning in the lipid metabolism increased after a HF meal^13,15^. In the present study, the expression of genes related to glucose uptake were decreased and those of glucose storage were increased comparing HF with LF consumption, which can be linked to the higher amount of fat in the HF meal and the increased amount of carbohydrates in the LF meal. In line with our results, in human muscle biopsies a shift in glucose metabolism from oxidation to storage was observed, when comparing HF with LF meal consumption^14^.

Besides the changes in expression of genes related to cholesterol ands lipid metabolism, which can easily be explained from a substrate point of view, the most apparent observation after consuming the HF meal was the upregulation of numerous gene sets and pathways associated with inflammation and immune function. Increased expression of cell adhesion molecules, cytokine and inflammatory responses as well as NF-κB and TNFα signaling were predominant. A number of studies looking in other tissues and species support our results. For example, in human PBMCs, the consumption of a HF breakfast increased the expression of IL-8 as compared with a LF breakfast^22^. Furthermore, inflammation was one of the most modulated biological processes after HF consumption in the intestines^21^ and adipose tissue^23^ of mice. In addition, in rat peripheral leukocytes a HF diet increased expression of genes related to leukocyte activation^24^. To visualize our findings, we presented the changes in all individual genes being part of the TNF signaling pathway (Fig. 3). This shows that a wide variety of inflammatory mediators are all upregulated during the HF meal as compared to the LF meal. This illustrates that it is not a single gene effect, but a general activation of inflammation processes activating cytokines, chemokines and adhesion molecules, which can all be linked to activated NF-kB signaling. This suggests that there is an ongoing activation of genes, already very early in the signaling cascade, which is confirmed by the activation of early upstream regulators like RELA (a subunit of NF-kB), MYD88, and the alarm cytokines IL1α and IL1β involved in inflammasome activation. The question is why HF diets activate these inflammatory systems. One explanation could be that saturated fatty acids, and particularly palmitic acid, which was present in significant amounts in the HF diets, is a potent substrate that is recognized by the pattern recognition receptor (PRR), Toll like receptor 4 (TLR4). Normally, TLR4 responds to lipopolysaccharide (LPS) molecules secreted by gram-negative bacteria. However, the LPS tail, which is the actual recognition site, is highly enriched in C16:0 molecules. Therefore, it has been suggested that other types of fat sources, such as long-chain polyunsaturated fatty acids, do not activate the NF-kB axis. Future studies are needed to confirm whether a HF-meal with a different fatty acid composition would have given different results.

Our results raise at least two additional questions. First, is the effect on inflammation a primary response to the intake of dietary fatty acids or is it a secondary response caused by enterocyte damage? Therefore, serum IFABP concentrations, a marker for enterocyte damage^25^, were determined, which were not different after the LF and HF meals, suggesting that the enterocytes were not damaged acutely after the HF meal. The second question is, whether the apparent increase in duodenal pro-inflammatory gene expression profiles translated into an inflammatory signature in postprandial serum samples. Our data clearly shows that none of the measured plasma markers differed after acute HF and LF consumption. This may indicate that the duodenal inflammatory signal needs longer than a few hours to translate into a systemic inflammatory response, or is not translated at all. Our results on plasma biomarkers are partly contradictory with other studies. Esser *et al*. found no changes in plasma CRP, ICAM-1, IL-6 and TNF-α, decreases in VCAM-1 and SAA, and an increase in IL-8 after an acute HF breakfast compared with an LF breakfast^16^. Nappo *et al*. found increased plasma TNF-α, IL6, ICAM-1 and VCAM-1 concentrations comparing HF with LF meal consumption^26^. It should be noted, however, that Esser *et al*. used a higher amount of fat^16^, while Nappo *et al*. studied diabetic patients^26^. Also, reported changes in plasma inflammation markers in response to HF and LF feeding have been very variable between studies^27^.

In conclusion, in healthy men, acute theobromine and fat consumption did not change apoA-I expression in the duodenum. Theobromine consumption inhibited intestinal gene expression related to glycogen and glucose breakdown, but did not change those related to lipid and cholesterol metabolism. Furthermore, HF intake activated expression of genes related to lipid and cholesterol uptake and transport and glucose storage, while it decreased those related to glucose uptake. Finally, microarray analyses suggested upregulation of inflammation in the duodenum after HF meal consumption, which was not translated into a systemic inflammatory response directly following a HF meal.

## Methods

### Study population and design

The design and results of the metabolic parameters of this double-blind crossover study have already been reported^17^. Briefly, ten apparently healthy men participated. During the screening visit, body weight, height and blood pressure were measured and a fasting blood sample was taken. Subjects were excluded when fasting serum total cholesterol concentrations ≥8.0 mmol/L or plasma glucose concentrations ≥7.0 mmol/L. After inclusion, all subjects participated in three test days, each separated by a one-week washout period. Two weeks before the start of the study, subjects were instructed to avoid products containing cocoa till the end of the study period. Also, the consumption of caffeine containing drinks was restricted to a maximum of 4 cups a day, since theobromine is a metabolite of caffeine. The study was conducted according the guidelines laid down in the Declaration of Helsinki. The study protocol was approved by the Medical Ethical Committee of the University Hospital Maastricht. All participants gave their written informed consent before entering the study. The study was registered on clinicaltrials.gov under study number NCT02085109, date of registration: 17-02-2014.

### Test days

To minimize differences in dietary intake before the three test days, all subjects were provided with a standard low fat dinner the evening before each test day, which consisted of a commercially available macaroni, 3 crackers, and a dairy drink. The next morning, subjects came to the University in fasting condition, which means that after dinner the preceding evening, they had not consumed any foods or drinks, except for water. To reduce physical activity as much as possible, participants arrived by public transport or car on the morning of the test day. After a 15 min rest, the first fasting blood sample was collected (T0) via an intravenous cannula inserted into the antecubital vein. Subjects were then asked to consume a shake within 10 min. Three different shakes were provided in random order. One shake was low-fat/high-carbohydrate (LF), one shake was LF enriched with 850 mg of theobromine (LF-TB), and one shake was high-fat/low-carbohydrate (HF) (Table 4). The theobromine powder (Fagron, Uitgeest, the Netherlands) was added as the final ingredient to the blender jar before it was thoroughly mixed with the LF shake. The volumes of the shakes were standardized with water. The difference between the HF and the LF shakes was an exchange between fat and carbohydrates, while the amount of proteins between the three shakes was comparable (Table 4). Shakes were prepared by the research dietician to blind both researchers and participants. After consumption of the shakes, the volunteers were not allowed to eat or drink anything except water for the next 5 hours. Other blood samples were taken at T = 30 (T30), T = 60 (T60), T = 90 (T90), T = 120 (T120), T = 180 (T180) and T = 240 (T240) min. Five hours after meal intake, duodenal biopsies were taken at the Department of Endoscopy. During duodenoscopy, no sedatives were given. Four duodenal mucosal tissue samples, just proximal of the ampulla of Vater, were taken using standard biopsy forceps. The diameter of the biopsies varied between 2.0 mm and 2.2 mm. After sampling, biopsies were immediately frozen in liquid nitrogen, stored at −80 °C and analyzed at the end of the study for gene expression profiles.

**Table 4:** Nutrient composition of the low-fat/high-carbohydrate (LF), LF with 850 mg theobromine (LF-TB) and high-fat/low-carbohydrate (HF) shakes.

Nutrient	LF	LF-TB	HF
Energy (kcal)	956	956	965
Protein (g)	19.4	19.4	17.9
(E%)^1^	8	8	7
Carbohydrates (g)	193.7	193.7	85.7
(E%)	81	81	35
Mono- and disaccharides (g)	144.9	144.9	45.6
Polysaccharides (g)	48.8	48.8	40.1
Total fat (g)	10.5	10.5	60.6
(E%)	10	10	56
Saturated fatty acids (g)	3.2	3.2	36.0
Monounsaturated fatty acids (g)	4.0	4.0	18.7
Polyunsaturated fatty acids (g)	1.1	1.1	4.1
Cholesterol (mg)	334	334	341
Theobromine (mg)	0	850	0

^1^E%: energy percent.

### Blood sampling and analysis

Blood was sampled in serum and EDTA-containing vacutainer tubes. Serum tubes were allowed to clot for 1 hour at 20 °C, followed by centrifugation at 1300 × g for 15 min at 20 °C. The EDTA tubes were placed on ice directly after sampling and centrifuged at 1300 × g for 15 min at 4 °C within 60 min after sampling. Serum and plasma aliquots were stored at −80 °C until analyses. All samples from one subject were analyzed within the same analytical run at the end of the study.

Because gene expression profiles clearly showed a more pro-inflammatory pattern after HF compared with LF consumption, concentrations of the inflammation and acute phase response markers: interleukin-6 (IL-6), interleukin-8 (IL-8), tumor necrosis factor-alpha (TNF-α), serum amyloid A (SAA), monocyte chemoattractant protein-1 (MCP-1), macrophage inflammatory protein 1 alpha (MIP-1α), and high sensitive C-reactive protein (hsCRP), were measured in plasma samples of the HF and LF test days (T0, T90 and T240). In addition we analyzed plasma concentrations of soluble intercellular adhesion molecule-1 (sICAM-1) and soluble vascular cell adhesion molecule-1 (sVCAM-1) to evaluate a possible cross-talk between an “inflamed” intestine and the vascular wall. For these analyses, a commercially available Multi Spot ELISA kit (Meso Scale Discovery, Rockville, MD, USA) was used. Finally, in the same plasma samples, intestinal fatty acid-binding protein (IFABP) concentrations, which is a marker for damaged enterocytes^25,28^, were measured using a sandwich ELISA (R&D, Oxon, United Kingdom) to evaluate whether one acute HF meal can already damage the enterocytes.

### Microarray processing and data analysis

Total RNA was extracted from one frozen duodenal mucosal biopsy using TRIzol reagent (Invitrogen, Breda, the Netherlands) and purified on columns using Qiagen RNeasy Micro Kit (Qiagen, Venlo, the Netherlands). Total RNA (100 ng) was labeled by Whole Transcript Sense Target Assay and hybridized to human whole-genome Affymetrix Gene 1.1 ST arrays targeting 19697 unique genes (Affymetrix, Santa Clara, CA). Microarray analyses were performed using MADMAX pipeline for statistical analysis of microarray data^29^. In short, microarrays were normalized with the robust multichip average method and probes were annotated as described^30,31^. This gene set was filtered on an expression of >10 on at least 5 arrays and measured with ≥5 probes. This filtered data set consisted of 10506 genes. Comparisons were made between the LF-TB and the LF meal and between the HF and the LF meal. Individual genes were defined as changed when comparison of the normalized signal intensities showed a p ≤ 0.05 in a 2-tailed paired intensity-based moderated t-statistics (IBMT) and a fold change of >1.2 or <−1.2 between the diets^32^. Further data analysis was performed on the filtered dataset with three different approaches i.e. Ingenuity Pathway Analysis (IPA), Upstream Regulator Analysis and Gene Set Enrichment Analysis (GSEA)^33^. Pathways were selected on a –log p-values of ≤1.3, which indicates a significant change of p ≥ 0.05 in that specific pathway comparing the LF-TB with LF diet or the HF with the LF diet. In the Upstream Regulator Analysis, Ingenuity software uses a curated database of interactions on the basis of the literature to link significant gene sets with upstream regulators. Significant linked gene sets were selected using a p-value of <0.05 for gene expression and a p-value of overlap of <0.05. A z-score above 1.5 indicates activation, whereas a z-score below -1.5 indicates inhibition of this upstream regulator. GSEA was performed on the unfiltered data set; gene sets were selected on a False Discovery Rate (FDR) q-value of <0.2 and were ranked on the Normalized Enrichment Score (NES). During microarray analysis we were especially interested in expression changes in apoA-I transcription and related pathways including the lipid, cholesterol and glucose metabolism, inflammation and the immune system.

### Statistical analysis

All data are presented as mean ± SD unless otherwise indicated. Differences in changes of inflammatory markers, endothelial function markers and IFABP between the HF and LF meal were evaluated with general mixed models with subject as random factor, diet and time as fixed factors and a diet*time interaction. If this diet*time interaction was not significant, it was omitted from the model. If the factor time was significant, time points were compared to baseline concentrations, using Bonferroni’s corrections for multiple comparisons. Results were considered to be statistically significant if p ≤ 0.05. All statistical analyses were performed using SPSS 20.0 for Mac (SPSS Inc., Chicago, IL, USA).

## Electronic supplementary material

Supplementary Information
