# Supplementary material for: The acute effects on duodenal gene expression in healthy men following consumption of a low-fat meal enriched with theobromine or fat
Source: Sci Rep. 2018 Jan 26;8:1700. doi: 10.1038/s41598-018-20068-y (PMC5785967; doi:10.1038/s41598-018-20068-y)
Supplement: Supplementary file 1 — Supplementary Information [file 41598_2018_20068_MOESM1_ESM.doc]

# Supplementary data

**The acute effects on duodenal gene expression in healthy men following consumption of a low-fat meal enriched with theobromine or fat**

Lotte Smolders^1^, Ronald P. Mensink^1^, Mark V. Boekschoten^2^, Rogier J.J. de Ridder^3^, Jogchum Plat^1^*

Figure 1 P-value distribution after A. adding theobromine (TB) to a low-fat/high-carbohydrate (LF) meal B. comparing high-fat/low-carbohydrate (HF) with LF consumption (*n* = 8)

A. B.


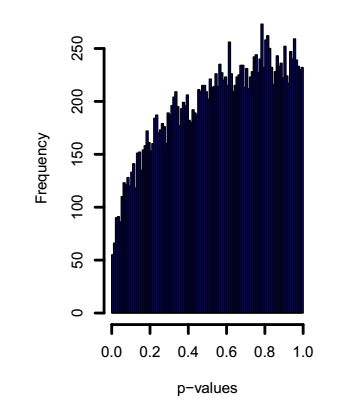

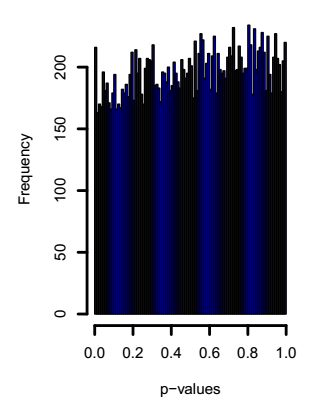


Table 1 Results of the upstream regulators after adding 850 mg of theobromine (TB) to a low-fat/high-carbohydrate (LF) meal (*n* = 8)

| **Upstream Regulator** | **Activation z-score** | **p-value of overlap** |
| --- | --- | --- |
| **U0126** | 1.71 | 0.00 |
| **streptozocin** | 1.72 | 0.00 |
| **LY294002** | 1.99 | 0.02 |
| **PD98059** | 2.21 | 0.01 |
| **SP600125** | 2.21 | 0.05 |
| **GATA4** | -1.50 | 0.02 |
| **calcitriol** | -1.50 | 0.00 |
| **lysophosphatidic acid** | -1.59 | 0.00 |
| **PDGF BB** | -1.60 | 0.03 |
| **decitabine** | -1.67 | 0.04 |
| **AGT** | -1.70 | 0.02 |
| **HNF4A** | -1.71 | 0.02 |
| **dexamethasone** | -1.89 | 0.00 |
| **FADD** | -1.94 | 0.01 |
| **Cg** | -1.94 | 0.02 |
| **MAP2K1/2** | -1.95 | 0.00 |
| **ATP** | -1.95 | 0.01 |
| **INS** | -1.95 | 0.04 |
| **SOX11** | -1.98 | 0.01 |
| **Insulin** | -2.08 | 0.04 |
| **aldosterone** | -2.17 | 0.01 |
| **EGF** | -2.19 | 0.02 |
| **butyric acid** | -2.19 | 0.01 |
| **Ca2+** | -2.19 | 0.04 |
| **Pkc(s)** | -2.19 | 0.05 |
| **GnRH analog** | -2.45 | 0.04 |
| **CREB1** | -2.88 | 0.01 |
| **Ins1** | -3.02 | 0.00 |

Table 2 Results of the GSEA comparing after adding 850 mg of theobromine (TB) to a low-fat/high-carbohydrate (LF) meal (*n* = 8)

| **Gene Set** | **NES** | **FDR-q value** |
| --- | --- | --- |
| **KINESINS** | 2.05 | 0.154 |
| **WP1897.REGULATION.OF.BETA.CELL.DEVELOPMENT** | 1.96 | 0.164 |
| **WP2739.AMYLOIDS** | 1.89 | 0.063 |
| **WP1842.KINESINS** | 1.88 | 0.068 |
| **KEGG_SYSTEMIC.LUPUS.ERYTHEMATOSUS** | 1.86 | 0.089 |
| **WP1874.NUCLEOSOME.ASSEMBLY** | 1.86 | 0.058 |
| **GLUCOSE.METABOLISM** | -1.96 | 0.138 |
| **WP716.VITAMIN.A.AND.CAROTENOID.METABOLISM** | -1.92 | 0.142 |

Table 3 Top 50 of the results of the upstream regulators after comparing high-fat/low-carbohydrate (HF) with low-fat/high-carbohydrate (LF) consumption (*n* = 8)

| **Upstream Regulator** | **Activation z-score** | **p-value of overlap** |
| --- | --- | --- |
| **Bleomycin** | 2.91 | <0.01 |
| **IL1B** | 2.82 | <0.01 |
| **E. coli B5 lipopolysaccharide** | 2.79 | <0.01 |
| **TNFSF11** | 2.73 | <0.01 |
| **L-glutamic acid** | 2.62 | 0.02 |
| **Pirinixic acid** | 2.57 | <0.01 |
| **LG100268** | 2.41 | <0.01 |
| **5-azacytidine** | 2.40 | 0.01 |
| **IL1A** | 2.35 | 0.01 |
| **Linoleic acid** | 2.31 | <0.01 |
| **PI3K (complex)** | 2.31 | <0.01 |
| **SREBF1** | 2.27 | <0.01 |
| **MYD88** | 2.24 | <0.01 |
| **Nicotinic acid** | 2.24 | <0.01 |
| **CSF1** | 2.22 | <0.01 |
| **Kainic acid** | 2.22 | 0.01 |
| **KLF15** | 2.20 | 0.01 |
| **IRS1** | 2.19 | <0.01 |
| **SCAP** | 2.18 | 0.01 |
| **Cocaine** | 2.17 | <0.01 |
| **AGN194204** | 2.16 | <0.01 |
| **Palmitic acid** | 2.06 | <0.01 |
| **Jnk** | 2.04 | <0.01 |
| **RELA** | 2.02 | 0.02 |
| **FABP2** | 2.00 | <0.01 |
| **Isoquercitrin** | 2.00 | <0.01 |
| **FDFT1** | 2.00 | <0.01 |
| **H2AFY** | 2.00 | 0.02 |
| **IL2RG** | 2.00 | 0.05 |
| **8-bromoguanosine 3'.5'-cyclic monophosphate** | 1.99 | 0.01 |
| **Tnf (family)** | 1.99 | 0.01 |
| **Advanced glycation end-products** | 1.98 | 0.02 |
| **BCR (complex)** | 1.98 | 0.04 |
| **CD5** | 1.98 | 0.05 |
| **PRKCE** | 1.98 | 0.04 |
| **Gsk3** | 1.98 | 0.03 |
| **CCL5** | 1.98 | 0.04 |
| **GCK** | -1.98 | <0.01 |
| **HAND1** | -1.98 | <0.01 |
| **HDL** | -1.98 | 0.02 |
| **ARNT** | -1.99 | <0.01 |
| **Dipyridamole** | -2.00 | <0.01 |
| **Calphostin C** | -2.00 | 0.02 |
| **Methotrexate** | -2.04 | <0.01 |
| **THRB** | -2.20 | 0.01 |
| **15-deoxy-delta-12.14 -PGJ 2** | -2.20 | 0.04 |
| **ABCB4** | -2.22 | <0.01 |
| **SB203580** | -2.28 | <0.01 |
| **IL10RA** | -2.39 | <0.01 |
| **GCG** | -2.42 | <0.01 |
| **U0126** | -2.48 | <0.01 |
| **NFE2L2** | -2.53 | <0.01 |
| **Bexarotene** | -3.14 | 0.01 |

Table 4 Top 50 of the results of the GSEA after comparing high-fat/low-carbohydrate (HF) with low-fat/high-carbohydrate (LF) consumption (*n* = 8)

| **Gene Set** | **NES** | **FDR-q value** |
| --- | --- | --- |
| **KEGG_MALARIA** | 2.17 | 0.017 |
| **WP143.FATTY.ACID.BETA.OXIDATION** | 2.09 | 0.025 |
| **CHEMOKINE.RECEPTORS.BIND.CHEMOKINES** | 2.08 | 0.020 |
| **COLLAGEN.DEGRADATION** | 2.05 | 0.022 |
| **WP2708.DEGRADATION.OF.COLLAGEN** | 2.03 | 0.024 |
| **WP2749.METABOLISM.OF.STEROID.HORMONES.AND.VITAMIN.D** | 1.94 | 0.057 |
| **REGULATION.OF.LIPID.METABOLISM.BY.PEROXISOME.PROLIFERATOR.ACTIVATED.RECEPTOR.ALPHA.PPARALPHA.** | 1.93 | 0.057 |
| **PPARA.ACTIVATES.GENE.EXPRESSION** | 1.92 | 0.058 |
| **WP368.MITOCHONDRIAL.LC.FATTY.ACID.BETA.OXIDATION** | 1.88 | 0.075 |
| **GENERATION.OF.SECOND.MESSENGER.MOLECULES** | 1.88 | 0.071 |
| **WP2406.CARDIAC.PROGENITOR.DIFFERENTIATION** | 1.84 | 0.108 |
| **KEGG_CELL.ADHESION.MOLECULES.CAMS.** | 1.82 | 0.113 |
| **KEGG_CYTOKINE.CYTOKINE.RECEPTOR.INTERACTION** | 1.82 | 0.110 |
| **WP325.TRIACYLGLYCERIDE.SYNTHESIS** | 1.82 | 0.106 |
| **WP2797.REGULATION.OF.LIPID.METABOLISM.BY.PEROXISOME.PROLIFERATOR.ACTIVATED.RECEPTOR.ALPHA.PPARALPHA.** | 1.81 | 0.104 |
| **WP530.CYTOKINES.AND.INFLAMMATORY.RESPONSE** | 1.80 | 0.103 |
| **KEGG_PPAR.SIGNALING.PATHWAY** | 1.80 | 0.104 |
| **PPARA_TARGETS** | 1.80 | 0.101 |
| **KEGG_INTESTINAL.IMMUNE.NETWORK.FOR.IGA.PRODUCTION** | 1.79 | 0.106 |
| **GLUCOSE.METABOLISM** | -2.29 | 0.001 |
| **WP1848.METABOLISM.OF.CARBOHYDRATES** | -2.17 | 0.006 |
| **KEGG_PROXIMAL.TUBULE.BICARBONATE.RECLAMATION** | -2.14 | 0.007 |
| **RIBOSOMAL.SCANNING.AND.START.CODON.RECOGNITION** | -2.05 | 0.020 |
| **KEGG_MINERAL.ABSORPTION** | -2.03 | 0.019 |
| **TRANSLATION.INITIATION.COMPLEX.FORMATION** | -2.02 | 0.018 |
| **ACTIVATION.OF.THE.MRNA.UPON.BINDING.OF.THE.CAP.BINDING.COMPLEX.AND.EIFS.AND.SUBSEQUENT.BINDING.TO.43S** | -1.94 | 0.045 |
| **KEGG_RIBOSOME** | -1.94 | 0.043 |
| **WP1889.PROCESSING.OF.CAPPED.INTRON.CONTAINING.PRE.MRNA** | -1.92 | 0.045 |
| **X3.UTR.MEDIATED.TRANSLATIONAL.REGULATION** | -1.88 | 0.068 |
| **GTP.HYDROLYSIS.AND.JOINING.OF.THE.60S.RIBOSOMAL.SUBUNIT** | -1.88 | 0.067 |
| **FORMATION.OF.THE.TERNARY.COMPLEX.AND.SUBSEQUENTLY.THE.43S.COMPLEX** | -1.86 | 0.068 |
| **L13A.MEDIATED.TRANSLATIONAL.SILENCING.OF.CERULOPLASMIN.EXPRESSION** | -1.85 | 0.072 |
| **TRANSLATION** | -1.85 | 0.070 |
| **GLUCONEOGENESIS** | -1.84 | 0.074 |
| **KEGG_FANCONI.ANEMIA.PATHWAY** | -1.83 | 0.078 |
| **WP1828.HEXOSE.TRANSPORT** | -1.83 | 0.073 |
| **KEGG_CARBOHYDRATE.DIGESTION.AND.ABSORPTION** | -1.83 | 0.070 |
| **PROCESSING.OF.CAPPED.INTRON.CONTAINING.PRE.MRNA** | -1.82 | 0.069 |
| **FORMATION.OF.A.POOL.OF.FREE.40S.SUBUNITS** | -1.82 | 0.066 |
| **WP2683.INFLUENZA.LIFE.CYCLE** | -1.82 | 0.064 |
| **KEGG_AMINOACYL.TRNA.BIOSYNTHESIS** | -1.82 | 0.061 |
| **MITOCHONDRIAL.TRANSLATION.ELONGATION** | -1.82 | 0.059 |
| **EUKARYOTIC.TRANSLATION.ELONGATION** | -1.81 | 0.061 |
| **CAP.DEPENDENT.TRANSLATION.INITIATION** | -1.81 | 0.058 |
| **BIOC_MTORPATHWAY** | -1.81 | 0.060 |
| **EUKARYOTIC.TRANSLATION.INITIATION** | -1.81 | 0.058 |
| **MRNA.SPLICING.MAJOR.PATHWAY** | -1.79 | 0.066 |
| **WP2773.DEGRADATION.OF.BETA.CATENIN.BY.THE.DESTRUCTION.COMPLEX** | -1.78 | 0.070 |
| **MITOCHONDRIAL.TRANSLATION** | -1.78 | 0.068 |
| **KEGG_TYROSINE.METABOLISM** | -1.78 | 0.067 |
|  |  |  |
